# A case of diphenhydramine intoxication showing prolonged false positive tricyclic antidepressant in the urine assay

**DOI:** 10.2478/abm-2023-0042

**Published:** 2023-08-12

**Authors:** Dongsun Kim, Hyun Young Shin, Bon D. Ku

**Affiliations:** Department of Neurology, Goyang Huymedi Hospital, Gyeonggi-Do 10564, Republic of Korea; Department of Medicine, Songpa-gu Health Center, Seoul 05552, Republic of Korea; Department of Neurology, Institute for Cognitive Intervention, International St. Mary's Hospital, Catholic Kwandong University College of Medicine, Incheon 22711, Republic of Korea

**Keywords:** ataxia, cross reactivity, diphenhydramine, tricyclic antidepressants, urine immunochromatographic assay

## Abstract

**Background:**

The urine immunochromatographic assay is a useful screening tool for patients suspected of acute drug intoxication in emergency conditions. Diphenhydramine intoxication shows symptoms similar to those of tricyclic antidepressant (TCA) intoxication.

**Case presentation:**

We examined a case of diphenhydramine intoxication showing cerebellar ataxia and prolonged false positive results for TCA in the urine. The urine TCA test showed persistently positive results even 60 h after the patient's initial drug screening. We observed negative conversion 90 h after the initial drug screening.

**Discussion:**

Considering the similarities of clinical symptoms between diphenhydramine and TCA intoxication, emergency physicians should consider the possibility of cross-reactivity in the diagnosis of a patient with unknown acute drug intoxication showing positive results of TCA immunochromatographic assay in the urine.

**Conclusion:**

The present case suggests that diphenhydramine overdose may cause cerebellar ataxia and show prolonged cross-reactivity as TCA in the urine.

Diphenhydramine is a commonly used over-the-counter preparation for the treatment of allergic rhinitis [1]. Diphenhydramine intoxication can result in confusion, tremor, athetosis, seizure, or cardiovascular collapse, which is similar to that of tricyclic antidepressant (TCA) intoxication [2, 3]. Recently, we examined a case of diphenhydramine intoxication showing cerebellar ataxia and prolonged false positive results for the TCA in the urine immunoassay. As far as we know, this is the first case report of prolonged cross-reactivity of diphenhydramine to the TCA in the urine immunoassay.

We obtained written informed consent from the patient to publish the present case report and associated images.

## Case presentation

A 59-year-old man visited our emergency department due to confusion, irritability, and tremulous movement after his intentional intake of unknown drugs. His unremarkable past medical history included depression or anxiety disorder. His initial vital signs were blood pressure at 104/86 mmHg; heart rate at 120 beats/min; respiratory rate at 20 breaths/min; and temperature at 36.5°C. Upon neurological examination, he showed confused mentality and agitated behavior. The patient's cranial nerves had preserved brain stem reflexes, with moderately dilated pupils. He showed preserved sensory, motor, and reflex functions, but with marked kinetic tremors and ataxia in both sets of limbs. We could not carry out a Romberg test or gait examination due to retropulsion and limb ataxia. His uncooperativeness made it difficult to obtain a precise history of the ingested drugs. The patient's confusion and limb ataxia continued for 30 min, despite the intravenous administration of lorazepam. His brain magnetic resonance imaging was unremarkable and electroencephalogram results showed intermittent generalized slowing without epileptic discharges. Laboratory examinations were unremarkable except for ethanol (73.7 mg/dL). For the causative drug screening, therefore, we performed urine drug tests covering methamphetamine, amphetamine, opiates, marijuana, cocaine, benzodiazepine, and TCA. No test indicated the presence of significant drug intoxication except for the TCA test using an AccusignTCA kit (Princeton Biomeditech Corp., NJ, USA) [4].

**Figure 1.**
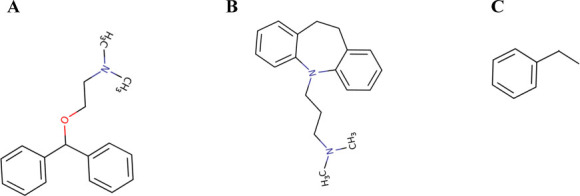
The chemical structure of dephenhydramine **(A)**, imipramine **(B)** and the common substructure of dephenhydramine and imipramine **(C)**. The similarities are as follow: AP Tanimoto: 0.421642, MCS Tanimoto: 0.2500, MCS Size: 8 and SMILES of common substructure: C(C)c1ccccc1. AP, atom pairs; MCS, maximum common substructure; SMILES, simplified molecular-input line-entry system.

We initially falsely diagnosed this patient with TCA intoxication by immunoassay and admitted him to the hospital for hydration, cardiac monitoring, and observation. Over the next 24 h, his consciousness gradually improved. From his detailed history, we learned that he had simultaneously ingested 40 tablets of diphenhydramine, with alcohol. The total amount of ingested diphenhydramine was 2000 mg (40 tablets, 50 mg/T). He recovered without specific therapeutic intervention, and a follow-up neurological examination, performed 36 h after admission, produced unremarkable findings, including in the Romberg and cerebellar function tests. We calculated the degree of structural similarities between dephenhydramine and TCA using the ChemMine tools [5]. We used imipramine as a TCA according to the AccusignTCA kit protocol. The simplified molecular-input line-entry system of dephenhydramine and imipramine was CN(C)CCOC(C1=CC=CC=C1)C1=CC=CC=C1, CN(C) CCCN1C2=CC=CC=C2CCC2=CC=CC=C12, respectively. The Atom Pairs (AP) and Maximum Common Substructure (MCS) were 0.421642 and 0.2500, respectively (**Figure 1**). The urine TCA test results persistently showed positive, even 60 h after his initial drug screening. We obtained a negative result at 90 h after his initial drug screening.

## Discussion

Diphenhydramine has a variety of neurochemical actions. It is a reversible, competitive inhibitor of histamine H_1_ receptors and also binds to muscarinic receptors or sodium channels [6]. Unlike its antihistaminergic effects, its anticholinergic or sodium channel blockade effects can occur as the diphenhydramine serum level increases [2, 3, 7]. Seizure is a recognized result of diphenhydramine's sodium channel blocking, and we consider the present case's generalized tremulous movement to be anticholinergic side effects of diphenhydramine overdose [2, 7, 8]. Diphenhydramine's anticholinergic side effects include dilated pupils, diplopia, dry mouth, flushed face, fever, urinary retention, and delirium [1, 7]. Cerebellar ataxia is a rare symptom of diphenhydramine intoxication. In the present case, the initial severe cerebellar ataxia may be related to the synergistic effects between diphenhydramine and alcohol. Alcohol is primarily metabolized in the liver by enzymes such as alcohol dehydrogenase and acetaldehyde dehydrogenase. Diphenhydramine is metabolized in the liver by enzymes such as cytochrome P450 enzymes. When alcohol and diphenhydramine are used together, they can compete for these liver enzymes, potentially leading to a slower metabolism of both substances. This can result in a longer retention time for diphenhydramine in the body more than usual [1]. The anti-cholinergic and sodium blockade effects of diphenhydramine can be enhanced by alcohol. Moser et al. [8] demonstrated that a diphenhydramine and alcohol combination significantly enhanced psychomotor performance in a man.

We used a one-step urine TCA test for screening. This is a simple immunochromatographic assay for the rapid detection of TCA [9]. A previous report of diphenhydramine producing a false positive result on the TCA test exists; Sorisky and Watson [10] reported the case of a serum, false positive result for TCA in a person who ingested 2,000 mg diphenhydramine and demonstrated that this false positive reaction had a dose-dependent nature. Farrell reported dimenhydrinate intoxication showing positive TCA assay in fluorescence polarization methods [11]. Dimenhydrate is a combination of diphenhydramine and chlorotheophylline. However, Sorisky did not perform a follow-up test on that case, and, thus, no knowledge exists regarding the duration of such false positive results in serum. In Farrell's case the gas chromatography mass spectrometry analysis performed after 6 h revealed positive diphenhydramine assay. In the present case, false positive reactions on this urine test persisted for at least 60 h. Although we did not perform quantitative testing for diphenhydramine, as far as we know, this is the first case report of prolonged cross-reactivity of diphenhydramine to the TCA in the urine immunoassay evaluating the structural similarities and Tanimoto coefficiency.

The exact mechanism whereby diphenhydramine shows a false positive result for TCA in the urine is still unclear. All drugs of abuse and toxicology screening immunoassays can be limited by false positivity caused by cross-reactivity from structurally related compounds [12]. According to the Accusign TCA kit, more than 150,000 ng/mL of diphenhydramine can produce a false positive result (0.3% cross-reactivity), and the urine TCA kit's cutoff value for nortriptyline is 1000 ng/mL [8]. Many TCAs and H_1_ antihistamines, such as diphenhydramine, have similar actions, for example, anticholinergic and serotonin uptake inhibition [13]. The similar ringed structures and metabolism between diphenhydramine and TCA can contribute to cross-reactivity when the concentration of diphenhydramine exceeds 100 μg/mL [13, 14]. In addition to diphenhydramine, a positive TCA result may be caused by other drugs such as carbamazepine, thioridazine, erphenazine, chlorpromazine, trimeprazine, cyproheptadine, cyclobenzaprine, and norcyclobenzaprine [15]. Due to diphenhydramine's dose-dependent toxicity, patients ingesting diphenhydramine at above 1000 mg dosages are at risk of developing severe intoxication symptoms and, therefore, need hospitalization [1, 3, 7, 9].

Regarding cross-reactivity between diphenhydramines and TCAs, it is important to note that not all drugs within these classes may exhibit the same level of cross-reactivity. Therefore, it would be necessary to consider all other TCA molecules and not make generalizations to calculate the degree of structural similarities between dephenhydramine and imipramine.

## Conclusion

Considering the similarities between diphenhydramine and TCA intoxication, emergency physicians should consider the possibility of cross-reactivity in the diagnosis of a patient with unknown acute drug intoxication. The present case suggests that diphenhydramine overdose may cause cerebellar ataxia and show prolonged cross-reactivity as TCA in the urine.
